# Acceptability of a first-line anti-tuberculosis formulation for children: qualitative data from the SHINE trial

**DOI:** 10.5588/ijtld.19.0115

**Published:** 2019-12-01

**Authors:** D. T. Wademan, L. Busakwe, T. J. Nicholson, M. van der Zalm, M. Palmer, J. Workman, A. Turkova, A. M. Crook, M. J. Thomason, D. M. Gibb, J. Seeley, A. Hesseling, G. Hoddinott, on behalf of the SHINE trial team

**Affiliations:** 1 Desmond Tutu TB Centre, Department of Paediatrics and Child Health, Stellenbosch University, Tygerberg, South Africa; 2 Medical Research Council Clinical Trials Unit at University College London, Institute of Clinical Trials and Methodology, London; 3 Department of Global Health, London School of Hygiene & Tropical Medicine, London, UK

**Keywords:** TB, paediatric, acceptability, palatability, fixed-dose combination formulations

## Abstract

**SETTING:**

We conducted a qualitative exploration into the palatability and acceptability of a novel fixed-dose combination (FDC) anti-tuberculosis drug. This study was nested in the SHINE (Shorter treatment for minimal TB in children) trial, which compares the safety and efficacy of treating non-severe drug-susceptible tuberculosis (TB) with a 6 vs. 4 months anti-tuberculosis regimen in children aged 0–16 years. Participants were recruited in Cape Town, South Africa.

**OBJECTIVE:**

To describe the palatability and acceptability of a FDC of rifampicin, isoniazid and pyrazin-amide among South African children and their caregivers in the SHINE trial.

**METHODS:**

We conducted 20 clinic observations of treatment administration, during which we conducted 16 semi-structured interviews with children and their caregivers. Data were organised thematically to report on experiences with administering and ingesting the FDC.

**RESULTS:**

Children and caregivers' experiences varied from delight to disgust. In general, participants said that the FDC compared favourably to other formulations. Pragmatic challenges such as dissolving the FDC and the time required to administer the FDC impeded caregivers' ability to integrate treatment into their daily routines. Drug manipulation was common among caregivers to improve TB treatment administration.

**CONCLUSION:**

This novel FDC appears acceptable for children, albeit with practical challenges to administration. Scale-up of FDC use should include supplementary intervention components to support caregivers.

## Abstract (FR)

**CONTEXTE:**

Nous avons réalisé une exploration qualitative de la palatabilité et de l'acceptabilité d'une nouvelle combinaison à doses fixes (FDC) de médicaments antituberculeux. Réalisée au sein de l'essai SHINE (*Shorter treatment for minimal TB in children*), qui compare la sécurité et l'efficacité du traitement d'une tuberculose (TB) pharmacosensible non grave avec un protocole antituberculeux de 6 mois au lieu de 4 mois chez des enfants âgés de 0–16 ans. Les participants ont été recrutés au Cap, Afrique du Sud.

**OBJECTIF:**

Décrire la palatabilité et l'acceptabilité d'une FDC de rifampicine, d'isoniazide et de pyrazinamide parmi des enfants Sud-Africains et leurs responsables dans l'essai SHINE.

**MÉTHODE:**

Nous avons réalisé 20 observations cliniques de l'administration du traitement, au cours desquelles nous avons conduit 16 entretiens semi structurés avec les enfants et leurs responsables. Les données ont été organisées par thème afin de rapporter les expériences des patients en matière d'administration et d'ingestion des FDC.

**RÉSULTATS:**

Les expériences des enfants et de leurs responsables ont varié de l'enchantement au dégoût. En général, les participants ont déclaré que les FDC se comparaient avantageusement aux autres formulations. Néanmoins, des problèmes pratiques comme la dissolution des FDC et le temps requis pour les administrer a entravé la possibilité d'intégrer le traitement dans la routine quotidienne. La manipulation des médicaments a été fréquemment faite par les responsables afin d'améliorer l'administration du traitement de TB.

**CONCLUSION:**

Cette nouvelle FDC semble acceptable pour les enfants, malgré des problèmes pratiques en matière d'administration. L'expansion du recours aux FDC devrait inclure des éléments supplémentaires d'intervention afin de soutenir les responsables.

## Abstract (SP)

**MARCO DE REFERENCIA:**

Se llevó a cabo una evaluación cualitativa del sabor y la aceptabilidad de una nueva asociación de dosis fijas (FDC) de fármacos antituberculosos. Fue este un estudio anidado en el ensayo SHINE (*Shorter treatment for minimal TB in children*), que comparaba la seguridad y la eficacia de tratar casos de tuberculosis (TB) no grave, normosensible, con esquemas de 6 meses y 4 meses en los niños de 0 a 16 años. Los participantes se ingresaron al estudio en Ciudad del Cabo en Suráfrica.

**OBJETIVOS:**

Describir la calidad del sabor y la aceptabilidad de una FDC de rifampicina, isoniazida y pirazinamida en los niños surafricanos y sus cuidadores que participaban en el ensayo clínico SHINE.

**MÉTODO:**

Se practicaron 20 observaciones clínicas de la administración del tratamiento, durante las cuales se realizaron 16 entrevistas semiestructuradas a los niños y sus cuidadores. Los datos se organizaron por temas, a fin de comunicar las experiencias en la administración y la ingestión de la FDC.

**RESULTADOS:**

Las experiencias de los niños y los cuidadores oscilaron entre el agrado y la repulsión. En general, los participantes afirmaron que la nueva formulación es al menos tan buena como las otras. Algunas dificultades prácticas como la disolución de la FDC y el tiempo necesario para administrarla obstaculizaron la capacidad de los cuidadores para integrar el tratamiento en sus rutinas cotidianas. Con frecuencia los cuidadores manipulaban el fármaco con el fin de mejorar la administración del tratamiento antituberculoso.

**CONCLUSIÓN:**

Esta nueva FDC parece aceptable para los niños, aunque persisten algunas dificultades prácticas de administración. Al ampliar la escala de utilización de la FDC es necesario incorporar a la intervención componentes adicionales que ayuden a los cuidadores.

ADMINISTERING MEDICATION to children can be problematic and challenging for both caregivers and health workers.^1^,^2^ Anti-tuberculosis treatment (ATT) for children is particularly difficult because regimens consist of multiple drugs given daily over a long period, typically 6 months, for drug-susceptible TB.^3^ Furthermore, research aimed at optimising ATT regimens and the development of anti-tuberculosis drugs has largely excluded children and the development of child-friendly formulations. ATT regimens and formulations for children are therefore often extrapolated from adult data.^4^ Extrapolations of this kind do not always result in optimal drug doses and child-friendly formulations. This can have an impact on treatment efficacy, increase the likelihood of toxicity, pill burden and costs to health services and families.^5^,^6^ Fixed-dose combination (FDC) formulations were designed to overcome some of these issues by including fixed ratios of multiple drugs in a single pill, and thus simplifying dosing regimens.^7^

FDCs offer several advantages, including a reduction in the chance of prescription errors due to the use of ‘weight-band’ dosing tablets, a reduction in the pill burden and a reduced likelihood of selective non-adherence.^8^ In 2014, the World Health Organization published revised recommendations for drug dosing in paediatric ATT regimens.^9^ The ratios of drugs in pre-existing FDCs were thus no longer optimal. To address these changes in treatment recommendations, new child-friendly FDCs that comply with the 2014 WHO guidelines, were developed.^10^ These FDCs are already being successfully implemented as part of national paediatric TB treatment programmes using simplified weight-band dosing in several countries across the world, including in some countries in Africa.^1^

As with other drugs designed for use in children, the new FDC is dispersible and has undergone significant taste-masking to increase ease of administration.^11–13^ Dispersible tablets offer the familiarity of administering liquid formulations to children, promising greater ease of administration, and potentially, improving adherence.^14^,^15^

Optimal implementation of FDCs requires the evaluation of their palatability and acceptability in the context of children's everyday lives, particularly with reference to caregivers and familial contexts.^14^,^16^ This includes evaluating the practical aspects of administration, the volume of medicine given and whether or not medication was mixed with food. Palatability is defined as “the organoleptic properties which includes smell, taste, aftertaste, dose volume or size and texture (mouthfeel)”, and is an important consideration for the overall acceptability among paediatric populations.^17–19^ Recent studies suggest that taste preferences may vary by age and between cultures and different settings, highlighting the need to show greater sensitivity to the immediate needs of local populations.^19–21^ Conducting palatability and acceptability evaluations in the populations for which the treatment is being developed is a crucial step in this process.^20^

We present the experiences of the first children and their caregivers to use the FDC in the SHINE (Shorter treatment for minimal TB in children) trial (ISRCTN 63579542) in a high TB burden setting.^13^ We suggest lessons learnt for optimising the administration of the FDC.

## METHODS

### Setting

The study was conducted in Cape Town, South Africa. All children included in this study were enrolled in the SHINE trial and had been recruited from Tygerberg Hospital (a tertiary referral hospital), Cape Town, South Africa, or the surrounding clinics. Cape Town has a high burden of paediatric TB, with children aged <15 years constituting 13% of the total TB cases notified, and with approximately 10–15% of children with confirmed TB being HIV-infected.^22^

### Study design

SHINE is a randomised, open-label, non-inferiority trial comparing the safety and efficacy of a 6- vs. 4-month ATT regimen using the WHO-recommended FDC in 1200 HIV-infected and non-infected children in South Africa, Zambia, Uganda and India.^23^ Children aged 0–16 years, weighing ≥3 kg, and with symptomatic non-severe TB, regardless of HIV status, were eligible to participate.

Children were randomised to receive either 4 or 6 months of ATT. Participants weighing <25 kg received ATT with the WHO-recommended paediatric FDC, while those weighing ≥25 kg received the non-dispersible adult formulation FDC. The paediatric FDC for the intensive phase of anti-tuberculosis treatment is a dispersible tablet containing three drugs: isoniazid (H) 50 mg +rifampicin (R) 75 mg + pyrazinamide (Z) 150 mg. Ethambutol was added as a separate tablet during the intensive phase if indicated, which in South Africa was only added in case of HIV-infected children. For the continuation phase, a two-drug FDC dispersible tablet containing H 50 mg+R 75 mg was provided. Each FDC tablet is approximately 100 mm × 50 mm in diameter and can be dissolved in as little as 5 ml of water.

Between September and December 2016, 28 participants were enrolled into the SHINE trial in Cape Town. During this initial trial recruitment phase, we conducted an exploratory sub-study on palatability and acceptability to describe patients' and caregivers' early experiences of the FDCs in South Africa.

### Sampling, recruitment and data collection

We conducted 20 days of clinic observation consisting of between one and four participants per observation day. Observations included some SHINE trial participants' first experience of the FDC at enrolment into the trial (often administered by a member of the trial team), as well as observations at follow-up visits with participants who had experienced 2, 4 and 8 weeks of home administration of the FDC. Participants were recruited to this sub-study during clinic observation days and were invited to participate in an interview at a convenient time and place. We sampled purposively to include participants with different familial experience of TB treatment. We continued data collection until we reached saturation in the experiences reported by participants. At each of the follow-up visits, we observed caregivers administering and participants ingesting the FDC. This allowed us to explore their adaptation to the treatment and differences in how they experienced palatability over time. We captured field notes recording these observations and completed semi-structured debriefing documents after data collection. All data were collected by experienced graduate sociobehavioural science researchers, led by DWand supervised by GH.

We conducted 14 semi-structured interviews with participants and caregivers at the clinic facility, and two semi-structured follow-up interviews at participants' homes. Although we did not formally interview any health workers, we had numerous informal conversations with health workers before, during and after observation days. In total, we spoke to 14 caregivers and children. In all cases, caregivers were women, usually with one child on treatment and children were often 0–5-years-old, with some 6–10-years-old. Interviews were conducted in Xhosa, Afrikaans or English using a semi-structured interview schedule. Discussions in these interviews focussed on the acceptability (including palatability) of the FDC, and any challenges to adherence for both participants and their caregivers. Specifically, we asked participants and their caregivers about 1) experiences of the FDC's palatability, 2) experiences of the FDC compared to any other experiences of ATT in their family, and 3) practical use of the FDC in the household context.

### Data analysis

Data analysis was an iterative process focussing on interactions between health workers, children, caregivers and researchers. After every observation and interview, the lead social scientist (DW) completed a structured reflexivity document. This document focusses specifically on health worker/participant/caregiver's understanding and use of the FDC, and its integration into everyday practices and processes (both at home and in the clinic).

All interviews were transcribed verbatim in Xhosa or Afrikaans, and then translated to English. A deductive thematic analytic approach was used to identify instances where caregivers and/or children spoke about their experience of administering and tasting the FDC.^24^ We first read through all interview transcripts and field notes to identify initial codes with a particular focus on palatability. Thereafter, codes were organised into themes. Objectives-based categories were refined through repeated analysis and re-reading of the data set to verify that interpretations fit the data. The analysis process and themes were cross-checked by the first, second and last authors to promote analytic reliability. Findings were member-checked with participants and health workers at subsequent interactions.

### Ethics statement and details of informed consent

The SHINE trial and sub-studies, including this nested palatability and acceptability evaluation, were approved by the Health Research Ethics Committee at Stellenbosch University Tygerberg, (M14/09/044), and by the Medicines Control Council of South Africa (#20150316), now called the South African Health Products Regulatory Authority (SAHPRA; Pretoria, South Africa). The trial, including this evaluation, is registered on the ISRCTN database (ISRCTN63579542). All adult participants (caregivers and health workers) observed and interviewed as part of the palatability and acceptability assessment provided written informed consent prior to participation. Caregivers' informed consent included their consent that the child TB patient in their care be included in observations and interview discussions.

## RESULTS

### Children and caregivers' experiences of the FDC's palatability

The majority of participants reported that the FDC was palatable, although there was some variability in responses. For some participants, the FDC was pleasant enough to be likened to a ‘treat’ for their child. For example, one mother spoke about how her 6-year-old daughter drank the treatment as if it were a ‘cool drink’ and said that when her daughter finished the course “she would see there's no more boxes [of treatment]” and “ask every day, ‘Mammie, where are my pills?’”.

However, most participants reported the FDC was still recognisably medicinal; for example, the mother of another 6-year-old girl explained that the FDC's appearance when dissolved, belied its taste. Both mother and daughter complained that the FDC was bitter, comparing it to a traditional aloe-based medicine (interview, 26 September 2016):

Yes, it's like *ukrakrayo* because it's bitter. But now because they are orange, I thought it was like Drink o'pop (powder-based drink concentrate). But when I tasted just a little like this [illustrates by bringing her little finger up to mouth]. I can't taste the bitterness [it was not immediately unpalatable to me]. But then when I ask my daughter, she says it's bitter, so I figured it probably is, because she eats it all the time [she knows what it tastes like more than I do]. (Mother of 6-year-old girl)

The excerpt illustrates that before the mother tasted the FDC she thought it would be pleasant, because of its colour. However, both mother and daughter (patient) were unpleasantly surprised that the FDC remains a medicine that tastes somewhat bitter.

In some instances of ingestion, children would reject administration outright. At these times, children appeared not to be responding to the FDC in isolation, but rather to their whole treatment experience and situational cues specific to that administration, for example, being tired. At these times, caregivers and health workers used a variety of techniques to overcome this response and encourage ingestion. Children would sometimes be willing to ingest the FDC with sufficient positive reinforcement of compliance—for example, being given high fives from caregivers and health workers for every sip swallowed. When ingestion met with a lot of resistance from the patient, caregivers often resorted to negotiation—for example, the mother mentioned above told her 6-year-old daughter “if you are a good girl and take your medicine then you can have a yoghurt”. However, in some cases, caregivers needed to use physical compulsion, restraining the child while forcing them to swallow the treatment. For one mother, this pattern of using physical restraint became the norm for ensuring administration for her 2-year-old daughter—often asking the girl's aunt to assist her while she compelled the child to ingest her treatment (interview, 13 October 2016a).

### Children and caregivers' comparisons to other anti-tuberculosis treatments

Despite the diverse reactions to the FDC's palatability, our findings suggest that the FDC compares favourably to the TB treatment caregivers have seen other people in their family use. An 8-year-old boy's caregiver compared the FDC regimen to her grandfather's ATT regimen, saying (interview, 08 September 2016):

Caregiver of 8-year-old boy: What can I say [laughing] those ones [the grandfathers' treatment regimen] would be difficult for the child.

Researcher: They would be difficult?

Caregiver of 8-year-old boy: Yes [laughs]

Researcher: Why? Why would [the] grandfathers ones [treatment regimen] be too difficult?

Caregiver of 8-year-old boy: They [the tablets] are a lot.

The caregiver suggests that children would struggle to consume many pills at a time. In addition to reducing the perceived pill burden, the FDC proved to have various other characteristics which eased administration. For example, its dispersibility in small volumes of water, relatively small size and ease of application to weight-bands—since almost all children received one or more whole, not partial tablets. One of the caregivers compared the FDC to her older niece's treatment regimen saying that, her niece's treatment “was big, it was a big pill not like hers [pointing to the FDC]”. She went on to cite the dispersible nature of the treatment as one of the reasons why she finds it a more acceptable regimen: “I think this one [the FDC] that dissolves in water is better for her [indicating to her daughter] and for me [as caregiver supporting her ingestion]”. Not only was the FDC easy to prepare and to administer, but its dispersibility also acted as a safeguard against spitting out the medicine.

Unlike other TB drugs, the FDC's dispersibility also gave caregivers the freedom to use various implements to administer treatment, including a spoon, small cup or syringe. As a result, caregivers were able to use very small volumes (as little as 5 ml) of water to prepare the FDC for administration. In the excerpt below, the caregiver illustrates how the FDC's small volume and adaptable administration allowed her to use a syringe to administer the treatment to her 20-month-old boy (interview, 19 September 2016b):

Mother of 20 month-old-boy: For that medication [the FDC], I took the pill I put in the cup, and then I put water and, then it melted. I take a syringe and then I give it [to him in the] syringe.

Researcher: And how many pills is he taking?

Mother of 20 month-old-boy: It's only one but now they say they gonna change it, it's gonna be two now.

Researcher: And then you use the syringe, do you have to hold him, is it difficult or?

Mother of 20 month-old-boy: I must hold him and then I give.

Researcher: Does he try to push it [the syringe] away with his hands?

Mother of 20 month-old-boy: No, he doesn't push it away.

While it is easier for children to refuse the spoon/cup, a syringe when placed inside the mouth induces the swallowing reflex and thereby ensures maximum ingestion. However, there are some concerns about the feasibility of cleaning the syringe sufficiently between administrations.

### Children and caregiver's practical use of the FDC in the household context

Caregivers still experience a number of challenges in administering the FDC in their homes and adopt innovative strategies to overcome these challenges. These challenges are also true to varying degrees for non-FDC formulations and other regimens. It should be noted that although the FDC is comparatively more palatable and its dispersibility improves ease of administration, this is not a panacea for TB treatment adherence among paediatric patients in South Africa. Some of the ongoing challenges suggest that overall acceptability of the FDC is not ideal.

Some caregivers combined the FDC with food—frequently yoghurt or porridge. Several of the caregivers used yoghurt to bribe their child to take the treatment. Although health workers' instructions were to refrain from administering the treatment with dairy products for fear of influencing the formulation's absorption, some caregivers insisted that the treatment be administered in or with yoghurt. For example, the mother of a 6-year-old girl said (interview, 26 September 2016) “it can be rather put in yoghurt, right? It could be better”. However, other caregivers said that their child preferred to swallow the tablet whole. This was particularly the case for older children who would otherwise complain about the FDC's taste—for example, the caregiver of a 10-year-old boy said, “it used to make him nauseous at that time [when he drank it dispersed in water]” (interview, 29 September 2016).

Another challenge revolved around the primary caregiver's ability to integrate timely administration of the FDC into everyday life. Caregivers often set aside 15–30 minutes each morning to administer treatment. One mother, whose 9-month-old boy resisted the treatment, described her experience with the treatment as follows (interview, 12 September 2016):

Mother of 9-month-old boy: I am just practicing now just before I go to work around half past five [in the morning] I must start dissolving them [the FDC] and then I leave them and I prepare everything else [like breakfast and getting dressed] and then at least by six o'clock I give it to him.

Another mother described how she too struggled to incorporate the treatment into her everyday schedule saying that the FDC stains clothing. To comply with the recommended morning administration, she woke up at 04:00 (am), undressed herself, undressed her daughter, and then spent 30–45 minutes coaxing her daughter to take the treatment before washing up and going to work. She undressed to avert the financial and time costs of having to clean and change both her own and her child's clothing each morning, should her daughter spit up the FDC. Children living with HIV would take their ART in the evening. Some caregivers misinterpreted the recommendation to administer TB treatment in the morning as a non-negotiable directive from health workers resulting in two burdensome drug administration events per day. Other caregivers decided to forego the recommended morning administration, relying rather on daily cues—such as eating dinner together as a family, or watching daily soap operas—to remind them to administer the FDC.

Caregivers also cited their child's physical and emotional state as hindering or helping administration. Other challenges included psychosocial factors (such as having to take time off work to attend clinic meetings), stigma (particularly for children going to school) and lack of knowledge/education or misperceptions about adverse effects, affected treatment administration and adherence. While none of our participants struggled to access safe, clean water, this may not be the case in other under-resourced countries.

## DISCUSSION

The FDC is part of a wave of new treatment regimens created to be more palatable and child-friendly, aiming to bring down barriers to long-term adherence and reduce mortality and the likelihood of drug resistance.^25–27^ Children and caregivers' experiences of the FDC's palatability ranged from delight to disgust. Because of its appealing colour and familiar presentation in soluble form, caregivers and children were surprised to find that the FDC was more bitter than expected. Caregivers were innovative in overcoming the challenges posed by poor palatability through positive reinforcement, bribery and, in some cases, physical compulsion. Additional strategies included administering the FDC in solid pill form rather than dissolving it or administering the FDC in/with solids and fluids (e.g., water, yoghurt, porridge, etc.). Different strategies appeared to work for different children of different ages, as caregivers managed incorporating the FDC into their everyday routine—which sometimes resulted in significant time and money costs.

Acceptability has previously been defined as the “overall ability of the patient and caregiver (defined as “user”) to use a medicinal product as intended (or authorised)”.^15^ More recently, Simon Bryson has endorsed a ‘patient-centred medicine’ definition of acceptability, which is medicine that “the patient will deem acceptable, to the extent that they will willingly participate in taking the medication on a routine basis, in chronic treatment”.^28^ Bryson goes on to explain that medicine should be considered ‘administration-friendly’, “which may be defined as one whose dose is easy to measure, and administer without modification”.^28^ While we believe our description of the ease with which caregivers and patients can prepare and administer the FDC meet Bryson's ‘administration-friendly’ criteria; both definitions of ‘acceptability’ belie the complexity of everyday life and the myriad pragmatic, financial and social challenges that caregivers face during the treatment process for children affected by TB.

Definitions of ‘acceptability’ for anti-tuberculosis drugs or regimens may differ in different sociocultural contexts and should therefore be investigated more systematically in diverse settings.^29^,^30^ Despite being given instructions on how to administer treatment, we observed that drug manipulation is common practice among caregivers who adapt and adopt different strategies to improve TB treatment administration to children. Further research is needed to confirm the pharmacokinetic implications of these real-life approaches to administration of anti-tuberculosis drugs (given with yoghurt or over a couple of hours). We recommend that drug designers better understand the practical issues in the administration of treatment in children before formulations are developed and taken to market—including acceptability for children and caregivers.

The limitations of this study are that the sample size was small and that data collection to the point of saturation across all experiences was unfeasible; these restrain us from being able to describe these complexities in more detail. We were also unable to conduct formal comparisons between childrens' prior experiences of ATT with the FDC used in the SHINE trial as none of the children included had received TB treatment before. Longer-term, more indepth, quantitative and qualitative research is needed to uncover the many factors involved in making treatment acceptable.

Recent research suggests that health workers should work with caregivers to establish tailored ingestion support processes that simultaneously ease administration and preserve drug bioavailability.^31^ We further recommend that scale-up of FDC use includes supplementary structured initial and ongoing education and adherence support to improve caregivers' ability to effectively manage administration.

## References

[1] Maleche-Obimbo E, Wanjau W, Kathure I (2015). The journey to improve the prevention and management of childhood tuberculosis: the Kenyan experience. Int J Tuberc Lung Dis.

[2] Stillson C H, Okatch H, Frasso R (2016). ‘That's when I struggle’. . . Exploring challenges faced by care givers of children with tuberculosis in Botswana. Int J Tuberc Lung Dis.

[3] Chiang S S, Roche S, Contreras C (2017). Barriers to the treatment of childhood tuberculous infection and tuberculosis disease: a qualitative study. Int J Tuberc Lung Dis.

[4] Nachman S, Ahmed A, Amanullah F (2015). Towards early inclusion of children in tuberculosis drugs trials: a consensus statement. Lancet Infect Dis..

[5] Gupta A, Khan M A (2013). Challenges of pediatric formulations: a FDA science perspective. Int J Pharm.

[6] Craig SR, Adams L V, Spielberg SP, Campbell B (2009). Pediatric therapeutics and medicine administration in resource-poor settings: a review of barriers and an agenda for interdisciplinary approaches to improving outcomes. Soc Sci Med.

[7] Collier R (2012). Reducing the “pill burden”. Can Med Assoc J.

[8] Graham S M, Grzemska M, Gie R P (2015). The background and rationale for a new fixed-dose combination for first-line treatment of tuberculosis in children. Int J Tuberc Lung Dis.

[9] World Health Organization (2014). Guidance for national tuberculosis programmes on the management of tuberculosis in children.

[10] World Health Organization (2016). New fixed-dose combinations for the treatment of TB in children. Towards zero deaths.

[11] Batchelor H K, Marriott J F (2013). Formulations for children: problems and solutions. Br J Clin Pharmacol.

[12] Ivanovska V, Rademaker C M, van Dijk L, Mantel-Teeuwisse AK (2014). Pediatric drug formulations: a review of challenges and progress. Pediatrics.

[13] Gibb D M, Crook A, Mugyenyi P (2015). Shorter treatment for minimal TB in children.

[14] Taneja R, Garcia-Prats A J, Furin J, Maheshwari H K (2015). Paediatric formulations of second-line anti-tuberculosis medications: challenges and considerations. Int J Tuberc Lung Dis.

[15] Mistry P, Batchelor H (2016). Evidence of acceptability of oral paediatric medicines: a review. J Pharm Pharmacol.

[16] Albanna A S, Smith B M, Cowan D, Menzies D (2013). Fixed-dose combination antituberculosis therapy: a systematic review and meta-analysis. Eur Respir J.

[17] Ternik R, Liu F, Bartlett J A (2018). Assessment of swallowability and palatability of oral dosage forms in children: report from an M-CERSI pediatric formulation workshop. Int J Pharm.

[18] Lin D, Seabrook J A, Matsui D M, King S M, Rieder M J, Finkelstein Y (2011). Palatability, adherence and prescribing patterns of antiretroviral drugs for children with human immunodeficiency virus infection in Canada. Pharmacoepidemiol Drug Saf.

[19] Baguley D, Lim E, Bevan A, Pallet A, Faust S N (2012). Prescribing for children - taste and palatability affect adherence to antibiotics: a review. Arch Dis Child.

[20] Angelilli M L, Toscani M, Matsui D M, Rieder M J, Deangelis C D (2000). Palatability of oral antibiotics among children in an urban primary care center. Arch Pediatr Adolesc Med.

[21] Smith C J, Sammons H M, Fakis A, Conroy S (2013). A prospective study to assess the palatability of analgesic medicines in children. J Adv Nurs.

[22] du Preez K, Schaaf H S, Dunbar R (2018). Complementary surveillance strategies are needed to better characterise the epidemiology, care pathways and treatment outcomes of tuberculosis in children. BMC Public Health.

[23] Chabala C, Turkova A, Thomason M J (2018). Shorter treatment for minimal tuberculosis (TB) in children (SHINE): a study protocol for a randomised controlled trial. Trials.

[24] Braun V, Clarke V (2006). Using thematic analysis in psychology. Qual Res Psychol.

[25] Abdulla S, Amuri B, Kabanywanyi A M (2010). Early clinical development of artemether-lumefantrine dispersible tablet: palatability of three flavours and bioavailability in healthy subjects. Malar J.

[26] Cram A, Breitkreutz J, Desset-Brethes S, Nunn T, Tuleu C (2009). Challenges of developing palatable oral paediatric formulations. Int J Pharm.

[27] Murray S, McKenna L, Pelfrene E, Botgros R (2015). Accelerating clinical drug development for children with tuberculosis. Int J Tuberc Lung Dis.

[28] Bryson S P (2014). Patient-centred, administration friendly medicines for children: an evaluation of children's preferences and how they impact medication adherence. Int J Pharm.

[29] Ukwaja K N, Modebe O, Igwenyi C, Alobu I (2012). The economic burden of tuberculosis care for patients and households in Africa: a systematic review. Int J Tuberc Lung Dis.

[30] Loveday M, Sunkari B, Master I (2018). Household context and psychosocial impact of childhood multidrug-resistant tuberculosis in KwaZulu-Natal, South Africa. Int J Tuberc Lung Dis.

[31] Richey R H, Craig J V, Shah U U (2013). MODRIC: manipulation of drugs in children. Int J Pharm.

